# Protocol for a systematic review, meta-analysis, and trial sequential analysis of clinical outcomes following accelerated versus conventional corneal collagen cross-linking for corneal ectasia

**DOI:** 10.1186/s13643-019-1004-x

**Published:** 2019-04-04

**Authors:** Siddharth Nath, Carl Shen, Alex Koziarz, Laura Banfield

**Affiliations:** 1 Division of Ophthalmology, Department of Surgery, McMaster University, Hamilton, Ontario Canada; 2 Department of Biochemistry and Biomedical Sciences, Michael G. DeGroote School of Medicine, Faculty of Health Sciences, McMaster University, 1200 Main Street West, Room 4N53, Hamilton, Ontario L8N 3Z5 Canada; 3 Faculty of Medicine, University of Toronto, Toronto, Ontario Canada; 4 Health Sciences Library, McMaster University, Hamilton, Ontario Canada

**Keywords:** Cross-linking, Accelerated, Corneal ectasia, Keratoconus, Systematic review

## Abstract

**Background:**

Collagen cross-linking (CXL) is an evolving procedure that enhances the biomechanical rigidity of the cornea and can slow or halt ectatic disease. CXL requires delivery of 5.4 J/cm^2^ of ultraviolet A (UVA) radiation to a cornea saturated with riboflavin in order to induce cross-link formation. The conventional CXL procedure achieves this fluence by exposing the cornea to a 3 mW/cm^2^ UVA lamp for 30 min; however, some surgeons have proposed accelerated protocols which achieve the same fluence in a shorter period of time by using a higher power light source. Whether accelerated protocols are as effective in arresting ectasia as the established conventional procedure remains unclear. Accordingly, this study will systematically review and synthesise the evidence on accelerated CXL and compare it to the conventional approach across an array of clinical outcomes.

**Methods:**

We will search 16 electronic databases, including MEDLINE, Embase, and the Cochrane Library, from inception to February 1, 2019. We will include all randomised controlled trials comparing accelerated and conventional CXL for any corneal ectatic disease. Two reviewers will independently screen search results to identify eligible articles, complete data collection, and conduct quality assessment. The quality of individual trials will be assessed using the Cochrane Collaboration’s Risk of Bias Assessment Tool. Our primary outcome will be the change in maximal keratometry (*K*_max_) at 12 months following treatment. Additional outcomes will include: incidence of disease progression, incidence of serious adverse events, as well as change in *K*_max_ at longest follow-up, mean stromal demarcation line depth, mean uncorrected distance visual acuity, mean corrected distance visual acuity, mean *K*_max_, mean endothelial cell density, mean central corneal thickness, mean spherical equivalent, mean intraocular pressure, and mean corneal power, at 12 months following treatment. We will calculate relative risks and 95% confidence intervals (CIs) for dichotomous outcomes and weighted mean differences and corresponding 95% CIs for continuous outcomes. Prespecified subgroup analyses will be performed to investigate heterogeneity. We will rate the overall quality of evidence using the Grading of Recommendations Assessment, Development, and Evaluation (GRADE) approach.

**Discussion:**

This review will provide a comprehensive evaluation of the evidence on accelerated and conventional CXL approaches and serve to inform clinical practice, medical device design, and future research. Evaluating variations of the CXL protocol aimed at reducing treatment duration is of critical importance and a prerequisite to expanding treatment availability to more patients.

**Systematic review registration:**

PROSPERO CRD42018104151

## Background

Corneal ectasias are progressive, degenerative ocular diseases which result from thinning, bulging, and structural changes in the peripheral, central, or paracentral cornea. These structural alterations distort vision, restrict lens fitting, and result in a substantial reduction in quality of life [1]. The most common corneal ectasia is keratoconus, which affects approximately 1 in 375 individuals [2]. Despite extensive research into the cellular, molecular, and genetic mechanisms underlying ectatic processes, the pathophysiology of these diseases remains unclear, and there is a paucity of disease-modifying treatments [3]. Although many patients with ectasia may initially be managed with spectacles or rigid gas permeable lenses, a subset can have continued disease progression that requires a corneal graft [1].

Corneal collagen cross-linking (CXL) is a minimally invasive procedure proposed in the late twentieth century as a potential disease-modifying intervention for keratoconus [4]. The aim of CXL is to increase the biomechanical rigidity of the cornea by inducing cross-links both within and between collagen fibrils in the corneal stroma. This is achieved by using riboflavin as a photosensitiser, which, upon exposure to ultraviolet A (UVA) light, induces cross-link formation via the natural lysyl oxidase pathway [4, 5]. These increased cross-links serve to stabilise the structure of the cornea and enhance its stiffness, thereby protecting against further degeneration. Animal studies investigating the effect of CXL on corneal structure have been largely supportive of this hypothesis, finding significantly increased biomechanical rigidity in treated corneas [6–8]. CXL was approved for treating keratoconus and other corneal ectasias in the early 2000s across Europe and in 2016 in the USA [9, 10].

The conventional approved CXL technique, commonly referred to as the ‘Dresden protocol’, begins with removal of the central 7 mm of corneal epithelium, followed by the administration of riboflavin both prior to and during exposure of the cornea to UVA light. A fluence of 5.4 J/cm^2^ was determined to be ideal for cross-link formation, and this protocol achieves it through exposure to a 3 mW/cm^2^ lamp for 30 min [9]. Multiple aspects of the conventional protocol have been modified by surgeons in efforts to refine the procedure and achieve improved safety and efficacy.

In particular, clinicians have focused on minimising the UVA light exposure time, in order to decrease the duration of the procedure. Harnessing the Bunsen-Roscoe law of reciprocity, which states that a biological effect is proportional only to the energy dose and not the method of energy delivery, some surgeons have introduced accelerated CXL [11]. This modified CXL protocol achieves the same 5.4 J/cm^2^ fluence of UVA radiation in a shorter duration of time by implementing a stronger UVA light source. The decrease in exposure time is inversely proportional to the increase in UVA light power (e.g., 3 mW/cm^2^ for 30 min translates to 9 mW/cm^2^ for 10 min). Shortening the treatment duration may improve patient comfort and minimise adverse events linked to prolonged corneal exposure [12]. Moreover, with a shorter procedure, clinicians can rapidly expand treatment availability to a greater number of patients and intervene to arrest progressive ectasia more swiftly.

### Why it is important to do this review

Although accelerated CXL is rooted in principles of physics and offers considerable theoretical benefits, it remains unclear whether it induces similar biomechanical changes in the cornea as the conventional protocol. Whether the increased UVA light power is associated with additional adverse events is also uncertain. Randomised trials and observational studies comparing accelerated and conventional CXL have shown promising results; however, the field has yet to reach a consensus [13, 14]. Thus, we will undertake a comprehensive systematic review and meta-analysis in order to evaluate the safety and efficacy of accelerated CXL in comparison to the conventional protocol.

## Methods

### Objective

This systematic review and meta-analysis will examine the safety and efficacy of accelerated CXL in comparison to conventional CXL across an array of clinically important outcomes.

### Registration

This study protocol has been developed in line with the Preferred Reporting Items for Systematic Reviews and Meta-Analysis Protocols (PRISMA-P) [15] statement and is registered with the International Prospective Register of Systematic Reviews (PROSPERO; registration number: CRD42018104151). We will conduct our systematic review and meta-analysis in accordance with the Preferred Reporting Items for Systematic Reviews and Meta-Analyses (PRISMA) [16, 17] guidelines and the Cochrane Collaboration’s Handbook for Systematic Reviews of Interventions [18]. If there are insufficient randomised trials (fewer than three) available to conduct a systematic review and meta-analysis, we will include observational studies and implement guidelines set forth by the Meta-analysis of Observational Studies in Epidemiology (MOOSE) consensus statement [19]. This protocol will be updated as required by PRISMA-P criteria [15], and amended versions will be made available on PROSPERO.

#### Literature search

We will conduct a detailed search of the following electronic databases: MEDLINE, Embase, Web of Science, Cochrane CENTRAL, Cochrane DSR, CINAHL, OpenGrey, metaRegister of Controlled Trials, LILACS, ClinicalTrials.gov, WHO Clinical Trials Database, WangFangData, CQVIP, INSPEC, COMPENDEX, and CNKI, from inception through February 1, 2019. We will use medical subject heading (Me*SH*) terms and keywords related to corneal ectasia and the cross-linking approach (accelerated or conventional) as well as clinical outcomes. Search strategies will be designed by a multidisciplinary research team composed of clinicians, researchers, and academic librarians with expertise in conducting systematic reviews. The proposed search strategy for the MEDLINE database is provided in Table 1. Our electronic search will be supplemented by manually screening the references of eligible articles, reviewing the proceedings of relevant meetings, and contacting clinical experts in the field. We will conduct our search without any restrictions on publication type, language, or time.

**Table 1:** Search strategy for the MEDLINE electronic database using the Ovid interface

Database	Search terms
MEDLINE 1946–present	1 Cross-Linking Reagents/ 2 CORNEA/ 3 cornea*.mp. 4 1 and (2 or 3) 5 ((crosslink* or cross link* or xlink* or x link*) adj3 (cornea* or collagen)).mp. 6 CCL.mp. 7 CXL.mp. 8 C3R.mp. 9 or/4–8 10 Dilatation, Pathologic/ 11 ectasia*.mp. 12 corneal diseases/ 13 cornea*.mp. 14 exp. Corneal Dystrophies, Hereditary/ 15 pellucid marginal degeneration.mp. 16 (fuch* adj2 atroph*).mp. 17 ((fuch* or groenouw*) adj2 dystroph*).mp. 18 KERATOCONUS/ 19 keratoconus.mp. 20 Corneal Endothelial Cell Loss/ 21 Corneal Wavefront Aberration/ 22 keratopath*.mp. 23 or/10–22 24 9 and 23 25 “Transendothelial and Transepithelial Migration”/ 26 trans-epitheli*.mp. 27 transepitheli*.mp. 28 epitheli*.mp. 29 (dresden or phototherapeutic or photo-therapeutic or iontophoresis or tight junctions).mp. 30 or/25–29 31 24 and 30 32 remove duplicates from 31 33 (accelerat* or nonaccelerat* or conventional or regular or rapid or flash-linking or KXL).mp. 34 24 and 33 35 remove duplicates from 34

#### Study selection

**Fig. 1:**
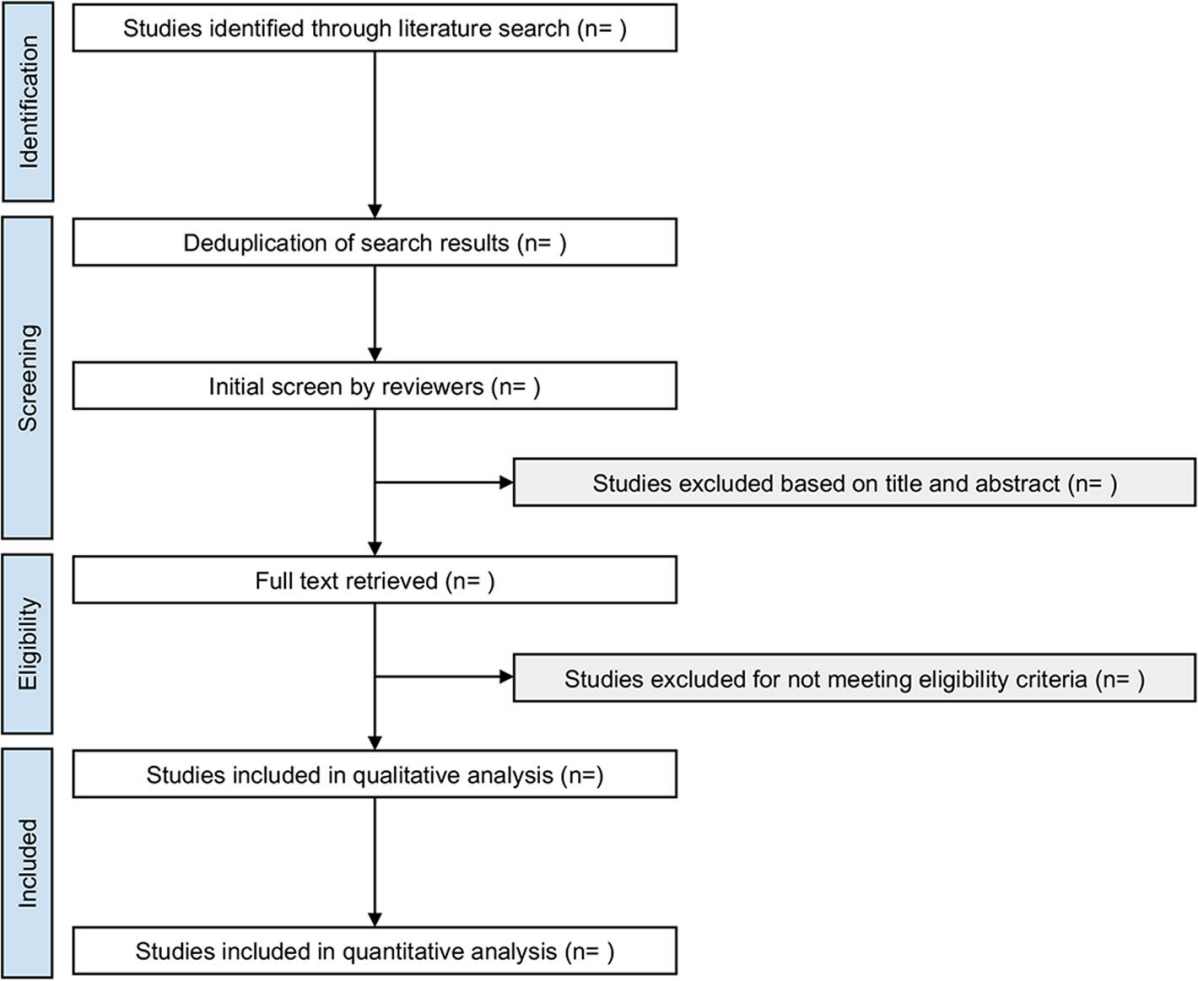
PRISMA Flow Diagram

Search results will be evaluated independently by two reviewers (SN, CS) against predefined eligibility criteria to identify relevant studies (Fig. 1). Results from all searched databases will be exported as .RIS, .XML, or .CIW files containing the complete reference, and EndNote X9 (Clarivate Analytics, Philadelphia, USA) software will be used for reference management. Reviewers will screen titles and abstracts against inclusion criteria, and full articles will be retrieved for all references that meet these criteria, or where there is any ambiguity. In cases of ambiguity, the complete report of the specific study will be screened independently by both reviewers in order to reach a judgement on inclusion. Disagreements between reviewers will be resolved by deliberation and consensus, and, if needed, including an impartial third reviewer (AK), or contacting the trial authors.

#### Eligibility criteria

Our inclusion criteria will be:

- *Population:* patients of any demographic undergoing CXL for treatment of corneal ectasia following refractive surgery, keratoconus, or pellucid marginal degeneration.
- *Intervention:* accelerated CXL
- *Control:* conventional CXL
- *Outcomes:* clinical outcomes such as change in maximal keratometry (*K*_max_) at 12 months after treatment (primary outcome), incidence of serious adverse events, as well as incidence of disease progression, change in *K*_max_ at longest follow-up, mean stromal demarcation line depth, mean *K*_max_, mean uncorrected distance visual acuity (UDVA), mean corrected distance visual acuity (CDVA), mean endothelial cell density, mean central corneal thickness, mean spherical equivalent, mean intraocular pressure, and mean corneal power, at 12 months following treatment.
- *Study design:* randomised controlled trials (including cluster trials and pilot studies) comparing accelerated and conventional CXL, with no publication type, language, or time restrictions. We will include both full articles and conference abstracts in the grey literature. Abstracts will be included only if they fulfil our eligibility criteria and if no subsequent study has been published. If we encounter duplicate studies, we will include only the report with the most current and complete data. We will exclude observational studies, unless insufficient (fewer than three) randomised trials are found, narrative reviews, systematic reviews, in vitro studies, letters to the editor and correspondences, and randomised trials examining only one CXL approach (accelerated or conventional) without a comparator. If insufficient (fewer than three) randomised controlled trials are available, we will include observational studies (prospective cohort only). Studies that examine different CXL approaches for indications other than corneal ectasia (e.g., infection) will also be excluded. We will record reasons for excluding studies.

#### Data management and collection

Data from included studies will be collected independently by two reviewers (SN, CS) and confirmed for accuracy by a third reviewer (AK). Prior to data extraction, the complete reports of all studies meeting our inclusion criteria will be collated, and reviewers will develop and pilot data extraction forms. For studies not published in English, the complete article will be translated into English and a clinical expert fluent in the original language of the study will be consulted. Discrepancies in data extraction will be resolved collaboratively by discussion amongst the two primary reviewers (SN, CS), conferring with an independent third reviewer (AK), or contacting the original trial authors. Where data in included studies is incomplete or ambiguous, we will contact study authors for further information and clarification.

We will extract data from eligible studies using forms with fields for the following: study first author, year of publication, journal of publication, language, study design, included centres, included countries, number of patients, number of males, number of females, recruitment period, eligibility criteria, method of randomisation, indication for CXL, number of patients in accelerated and conventional groups, age of patients in accelerated and conventional groups, procedure for follow-up, number of patients with disease progression 12 months following CXL in accelerated and conventional groups, *K*_max_ before CXL in accelerated and conventional groups, *K*_max_ 12 months after CXL in accelerated and conventional groups, *K*_max_ at longest follow-up after CXL in accelerated and conventional groups, UDVA before CXL in accelerated and conventional groups, UDVA 12 months after CXL in accelerated and conventional groups, CDVA before CXL in accelerated and conventional groups, CDVA 12 months after CXL in accelerated and conventional groups, central corneal thickness before CXL in accelerated and conventional groups, central corneal thickness 12 months after CXL in accelerated and conventional groups, endothelial cell density before CXL in accelerated and conventional groups, endothelial cell density 12 months after CXL in accelerated and conventional groups, intraocular pressure before CXL in accelerated and conventional groups, intraocular pressure 12 months after CXL in accelerated and conventional groups, corneal power before CXL in accelerated and conventional groups, corneal power 12 months after CXL in accelerated and conventional groups, spherical equivalent before CXL in accelerated and conventional groups, spherical equivalent 12 months after CXL in accelerated and conventional groups, stromal demarcation line depth after CXL in accelerated and conventional groups, method for epithelium removal, method for transepithelial riboflavin application, postoperative pain following accelerated and conventional CXL, time to best CDVA following accelerated and conventional CXL, time to best UDVA following accelerated and conventional CXL, power of UVA light in accelerated group, UVA light exposure time in accelerated group, incidence of significant complications (e.g., corneal melt, persistent epithelial defects, scarring, and persistent stromal haze) after accelerated and conventional CXL, and protocol preference as reported by authors.

#### Risk of bias in individual studies

All included trials will be assessed using the Cochrane Collaboration’s Risk of Bias Assessment Tool by two independent authors (SN, CS) [20]. Studies will be assessed to determine the risk of selection, performance, detection, attrition, reporting, and other biases. Discrepancies in quality assessment will be resolved collegially by deliberation and consensus, and consultation with an impartial third reviewer (AK). If there is insufficient information available to make a judgement on risk within an individual domain, we will rank that domain as ‘unclear’ and the original trial authors will be contacted for further information. Studies with one or more domains assessed to be ‘high risk’ will be categorised as having an overall high risk of bias.

#### Definition of outcomes

Our primary outcome will be the change in *K*_max_ (in dioptres, D) over 12 months. Additional outcomes shall be the following: incidence of disease progression (defined as an increase of the *K*_max_ by ≥ 1.0 dioptres (D) over 12 months) after treatment, as well as change in: mean UDVA (in logMAR) at 12 months following treatment, mean CDVA (in logMAR) at 12 months following treatment, mean endothelial cell density (in cells/mm^2^) at 12 months following treatment, mean *K*_max_ (D) at longest follow-up after treatment, mean stromal demarcation line depth (in μm) after treatment, mean central corneal thickness (in μm) at 12 months following treatment, mean spherical equivalent (D) at 12 months following treatment, mean intraocular pressure (in mmHg) at 12 months following treatment, mean corneal power (D) at 12 months following treatment, and the mean *K*_max_ (D) at 12 months after treatment. We will also examine the incidence of serious adverse events (e.g., corneal melt, persistent epithelial defects, scarring, and persistent stromal haze).

#### Data synthesis

Analyses will be conducted by the intention-to-treat principle using pooled study-level data. For dichotomous outcomes, such as incidence of ectatic disease progression and incidence of serious adverse events, we will summarise our analyses by calculating the relative risk (RR) with corresponding 95% confidence interval (CI). For continuous outcomes, such as change in *K*_max_, mean *K*_max_, mean UDVA, mean CDVA, mean central corneal thickness, mean stromal demarcation line depth, mean endothelial cell density, mean intraocular pressure, mean corneal power, and mean spherical equivalent, at 12 months following treatment, we will compute the weighted mean difference (MD) with 95% CI. Moreover, we will determine pooled estimates of all incidences across studies for the accelerated group, and then separately for the conventional group. The DerSimonian and Laird random effects model will be used to conduct our meta-analysis, and weights will be calculated using the inverse variance method [21]. The threshold for type I error for statistical significance shall be *α* = 0.05. Between-study heterogeneity will be assessed using Cochran’s *Q* test and quantified by the *I*^2^ statistic, with *I*^2^ values in excess of 25%, 50%, and 75%, graded as low, moderate, and high heterogeneity, respectively [22]. Publication bias will be examined qualitatively by visual inspection of funnel plot symmetry and quantified by Begg and Mazumdar’s [23] and Egger’s tests [24].

We will undertake prespecified subgroup analyses to investigate whether covariates exist and to examine heterogeneity in our primary outcome. Analyses will be performed for subgroups stratified by patient age and sex, CXL technique (transepithelial versus epithelium-off), severity of disease at time of treatment, and study risk of bias (low versus high). In addition, we will conduct a sensitivity analysis by performing trial sequential analysis (TSA) [25, 26]. TSA adjusts for the risk of type I error and constructs monitoring boundaries to determine whether an intervention is beneficial, harmful, or futile in comparison to control. We will perform TSA with an overall power of 80% and type I error risk of 5%.

The quality of available evidence will be summarised using the Grading of Recommendations Assessment, Development, and Evaluation (GRADE) approach [27]. Evidence will be assessed for the domains of risk of bias, consistency, precision, reporting bias, directness, and other bias. The evidence for each outcome will be ranked as being of high, moderate, low, or very low quality in accordance with criteria set by the GRADE working group.

All statistical analyses will be conducted using Comprehensive Meta-analysis v.3.3.070 (Biostat, Englewood, New Jersey, USA), and TSA will be performed using Trial Sequential Analysis v0.9.5.10 Beta (Copenhagen Trial Unit, Copenhagen, Denmark).

## Discussion

The results of this review are expected to considerably inform clinical practice, future research, and the management of patients with ectatic disease. By comprehensively examining the evidence on accelerated and conventional CXL approaches, this study is expected to influence how clinicians perform this evolving treatment and how UVA cross-linking devices are engineered and, potentially, improve patient outcomes. Moreover, through analysing the safety and efficacy of accelerated CXL, this study may incite changes leading to shorter operative times and increased treatment availability. Findings from this study will be submitted to a peer-reviewed journal for publication and will be presented at conferences and seminars.

## Abbreviations

CDVA: Corrected distance visual acuity
CI: Confidence interval
CXL: Corneal collagen cross-linking
GRADE: Grading of Recommendations Assessment, Development, and Evaluation
*K*_max_: Maximal keratometry
MD: Mean difference
Me*SH*: Medical subject heading
PRISMA: Preferred Reporting Items for Systematic Review and Meta-Analyses
PRISMA-P: Preferred Reporting Items for Systematic Review and Meta-Analysis Protocols
PROSPERO: International Prospective Register of Systematic Reviews
RR: Relative risk
TSA: Trial sequential analysis
UDVA: Uncorrected distance visual acuity
UVA: Ultraviolet A
